# Supplementary material for: Biofunctionality of Enzymatically Derived Peptides from Codfish (Gadus morhua) Frame: Bulk In Vitro Properties, Quantitative Proteomics, and Bioinformatic Prediction
Source: Mar Drugs. 2020 Nov 27;18(12):599. doi: 10.3390/md18120599 (PMC7759894; doi:10.3390/md18120599)
Supplement: Supplementary file 1 [file marinedrugs-18-00599-s001.zip › marinedrugs-1006687-SI-proofed/Supporting Information_SGR-Ali_SGR.docx]

Supplementary Materials

Biofunctionality of Enzymatically Derived Peptides from Codfish (*Gadus morhua*) Frame: Bulk in Vitro Properties, Quantitative Proteomics, and Bioinformatic Prediction

Ali Jafarpour ^1,^*^,†^, Simon Gregersen ^2,^*^,†^, Rocio Marciel Gomes ^1^, Paolo Marcatili ^3^, Tobias Hegelund Olsen ^3^, Charlotte Jacobsen ^1^, Michael Toft Overgaard ^2^, and Ann-Dorit Moltke Sørensen ^1^

^1^ Research Group for Bioactives-Analysis and Application, Division of Food Technology, National Food Institute, Technical University of Denmark, Kgs. Lyngby, 2800, Denmark; s182389@student.dtu.dk (R.M.G.), chja@food.dtu.dk (C.J.), adms@food.dtu.dk (A.-D.M.S.)

^2^ Section for Biotechnology, Department of Chemistry and Bioscience, Aalborg University, Aalborg, 9220, Denmark; mto@bio.aau.dk

^3^ Department of Health Technology, Technical University of Denmark, Kgs. Lyngby, 2800, Denmark; pamar@dtu.dk (P.M.), tobhe@dtu.dk (T.H.O.)

***** Correspondence: alijaf@food.dtu.dk (A.J.); sgr@bio.aau.dk (S.G.).

**^†^ T**hese authors contributed equally: Ali Jafarpour (A.J.) and Simon Gregersen (S.G.).

**Table S1.** Combinatorial space of peptides as a function of length for peptides consisting of merely the 20 naturally occurring and unmodified L-amino acids (N=20) and for peptides including the default variable modifications from MaxQuant i.e. Met oxidation and N-terminal peptide alkylation (N=21+1).

| **Amino Acids** | **Peptides (N=20)** | **Peptides (N=21+1)** |
| --- | --- | --- |
| 1 | 20 | 22 |
| 2 | 400 | 462 |
| 3 | 8000 | 9702 |
| 4 | 1.60E+05 | 2.04E+05 |
| 5 | 3.20E+06 | 4.28E+06 |
| 6 | 6.40E+07 | 8.99E+07 |
| 7 | 1.28E+09 | 1.89E+09 |
| 8 | 2.56E+10 | 3.96E+10 |
| 9 | 5.12E+11 | 8.32E+11 |
| 10 | 1.02E+13 | 1.75E+13 |
| 11 | 2.05E+14 | 3.67E+14 |
| 12 | 4.1E+15 | 7.71E+15 |
| 13 | 8.19E+16 | 1.62E+17 |
| 14 | 1.64E+18 | 3.4E+18 |
| 15 | 3.28E+19 | 7.14E+19 |
| 16 | 6.55E+20 | 1.5E+21 |
| 17 | 1.31E+22 | 3.15E+22 |
| 18 | 2.62E+23 | 6.61E+23 |
| 19 | 5.24E+24 | 1.39E+25 |
| 20 | 1.05E+26 | 2.91E+26 |
| 21 | 2.1E+27 | 6.12E+27 |
| 22 | 4.19E+28 | 1.29E+29 |
| 23 | 8.39E+29 | 2.7E+30 |
| 24 | 1.68E+31 | 5.67E+31 |
| 25 | 3.36E+32 | 1.19E+33 |
| 26 | 6.71E+33 | 2.5E+34 |
| 27 | 1.34E+35 | 5.25E+35 |
| 28 | 2.68E+36 | 1.1E+37 |
| 29 | 5.37E+37 | 2.32E+38 |
| 30 | 1.07E+39 | 4.86E+39 |

**Table S2.** Sequences, relative intensity, and predicted emulsifying activity in α-helical, β-strand, and γ (axial amphiphilicity) conformation for the 100 peptides with highest relative intensity (PCL > 6) from enzymatically derived FPH from minced cod frame (MCF) and heated cod meat (HCM).

| **MCF** | | | | | | | | | | | | | | | | |
| --- | --- | --- | --- | --- | --- | --- | --- | --- | --- | --- | --- | --- | --- | --- | --- | --- |
| **Neut.** | **Rel. Int. (%)** | **Emulsifying Scores of different possible conformation** | | | | **Alc** | **Rel. Int. (%)** | Emulsifying Scores of different possible conformation | | | Neut&Alc | Rel. Int. (%) | Emulsifying Scores of different possible conformation | | |  |
|  |  | **α** | **β** | **γ** |  | |  | α | β | γ |  |  | α | β | γ |  |
| LQGEVEDLMVDVERANG  LEQQVDDLEGSLEQEKK  IITNWDDMEK  VQHELEEAEERADIAETQVNK  LTKLEEAEKAADESERGMK  LEDQLSEIKAKSDENARQ  LEKSYELPDGQVIT  VAPEEHPTL  IIDQNRDGIISKDDLRD  LDDLQAEEDKVNT  LEKTIDDLEDELYAQK  LKGTEDELDKYSEALKDAQEKLE  LTEEMASQDESVAK  LKGADPEDVIVAA  LADWKQKYEEGQAELEGSLKEARS  SKYETDAIQRTEELEESKKK  LKAGDSDGDGAIGVDEWAV  LTDAETKAF  LKAGDSDGDGAIGVDEWAVLVKA  VDDIIQTGVDNPGHPFIMT  VIDQDKSGFIEEDELKLF  VMVGMGQKDSYVGDEAQSKRG  LDFENEMAT  IDDLEDELYAQK  VQGEVDDSVQEARNAEEKAKKA  ARIEELEEELEAERA  LVQVQGEVDDSVQEARNAEEKAKKA  ARIEELEEELEAERAAR  VASIDDKEELDATDAAIDILG  FVIDQDKSGFIEEDELK  LDDAVRAAEDLKEQAAM  VAGDEESYEVFKD  LEKEKSEYKMEIDDLSSNMEAVSKAKGN  ILDPEATGSIKKEF  LEDECSELKKDIDDLELT  LTENGEFGRQLEEKEA  ISDLTEQLGETGKS  LVEEELDRAQER  SAPKIPDGEKVDFDDIQKKRQNKD  ALEEAEGTLEHEESKLLR  VLSGGTTMYPG  LDDVIQTGVDNPGHPFIMT  ILEEECMFPKASDATFKAKL  VALDFENEMAT  LAQESIMDLENDKQQSDEKLKKKDFETSQ  ISEELDHALNDMTSI  ADWKQKYEEGQAELEGSLKEARS  LNVKNEELEAMVKE  ADSVAELGEQIDNLQR  LESDLVQVQGEVDDSVQEARNAEEKAKK  IGMESAGIHET  VRDLESEVDNEQRRGAEA  GPSGAPGPVGPAGKTGDRGETGPAG  VMDLENDKQQSDEKLKKKDFETSQL  IVLDSGDGVTHNVPVYEGY  LEKSYELPDGQVITIGNERFRCPET  VIDQDKSGFIEEDELK  FTPDQMEDYREAFG  LAQESIMDLENDKQQSDEKLKKKDFETSQL  VAEQELIDASERVGL  LEKEKSEYKMEIDDLSSNMEA  LKAGDSDGDGAIGVDEWA  LDDAIRAADDLKEQAAM  DWKQKYEEGQAELEGSLKEARS  AAGPAGPSGPRGPAG  LDDAVRAAEDLKEQA  IEELEEELEAERAAR  VETEKTEIQSALEEAEGTLEHEESK  IIAPPERKYS  MEGDLNEMELQ  LKREEADAKKKMEEDAKKKSA  IDASERVGL  IEELEEELEAERA  LQEAEEQIEAVNSKCAS  LVIIESDLERTEERAE  IGMESAGIHETAYNS  VDDIIQTGVDNPGHPF  VAQWRSKYETDAIQRTEELEESKKK  LVEEELDRAQERL  MEIDDLSSNMEAVSKAKGN  LEEISERLEEAGGATSAQ  MAEELKKEQDTSSHLERMKKN  LEKSESIDDMIPAQK  AMKDEEKMELQEIQ  LDKNKDPLNDSVVQ  GEVDDSVQEARNAEEKAKKA  ADGNVGPAGPAGPLG  VIIESDLERTEERAE  ILEEECMFPK  ILADADCAAA  LAEKDEEMEQIKRNSQR  LSKIEDEQSLGAQ  FEQSQIQEYKEAFT  FSGDEEFPDLS  LKETTERLEDEEEINAE  ILDPEATGSIKKE | 1.94  1.92  1.78  1.39  1.17  1.00  0.92  0.90  0.86  0.81  0.78  0.77  0.76  0.76  0.71  0.69  0.69  0.65  0.61  0.59  0.57  0.57  0.54  0.54  0.54  0.53  0.53  0.52  0.52  0.52  0.51  0.49  0.48  0.47  0.47  0.45  0.43  0.42  0.41  0.41  0.39  0.39  0.38  0.37  0.36  0.35  0.35  0.35  0.34  0.33  0.33  0.33  0.32  0.32  0.32  0.32  0.32  0.32  0.31  0.31  0.31  0.31  0.31  0.30  0.30  0.29  0.29  0.29  0.29  0.28  0.28  0.28  0.27  0.27  0.27  0.27  0.26  0.26  0.26  0.26  0.26  0.25  0.25  0.25  0.25  0.24  0.24  0.24  0.24  0.23  0.23  0.23  0.23  0.22  0.22  0.22 | 1.38  2.18  0.75  2.21  1.53  1.54  -0.55  -0.18  -0.51  0.55  1.51  2.96  1.01  -1.53  0.70  0.71  0.26  0.43  -0.98  0.89  -1.61  -0.69  0.10  0.45  2.82  -0.65  2.64  -1.60  0.00  -1.60  2.43  0.01  0.79  -0.52  1.84  0.10  2.57  1.58  -0.05  1.83  -0.22  0.83  -1.06  0.13  1.01  2.99  0.32  0.67  2.85  2.83  -0.62  2.15  -0.09  0.66  0.37  0.47  -1.23  0.24  1.05  0.21  0.33  0.26  2.44  0.61  -0.56  3.07  -0.70  1.33  -0.59  -0.76  0.20  0.02  0.22  1.80  0.57  -0.01  2.03  0.86  2.17  0.60  1.34  0.81  -0.16  -0.18  1.41  2.44  -0.75  0.24  -0.43  -0.78  1.24  0.01  -0.21  -0.08  0.74  -0.63 | -0.45  -0.87  -1.28  -0.41  -0.55  0.10  -0.43  -0.07  -0.56  -1.02  -0.63  0.22  -0.17  -0.19  -0.08  -0.69  -0.72  -0.14  -1.16  0.34  -0.47  -0.91  1.04  0.14  1.33  -0.93  1.54  -0.13  -0.74  -0.95  1.34  -1.11  -0.30  1.36  -0.42  0.33  -0.34  -1.28  -0.25  -0.16  -0.94  0.33  1.50  1.18  -1.19  0.64  -0.47  -1.33  0.23  0.55  -0.94  0.79  -0.27  -0.93  0.67  -1.10  -0.56  -1.18  -1.05  0.78  -1.03  -0.18  1.38  -0.25  -0.75  1.49  0.82  -1.19  -0.62  -1.27  0.62  2.31  0.05  -1.25  -1.30  -1.04  0.24  -0.28  -0.82  0.20  -1.22  -0.44  0.29  -0.52  -0.79  0.52  -0.32  -0.75  -0.67  0.73  -0.99  0.81  -0.81  -0.84  0.96  1.04 | 0.55  1.26  2.95  0.64  1.15  1.33  0.82  1.04  0.70  1.17  0.12  0.47  0.11  2.78  0.43  1.57  1.09  -0.44  1.53  0.07  -0.33  2.46  0.59  1.00  1.45  0.54  2.42  0.76  1.40  1.26  0.27  0.93  0.66  -0.24  0.82  0.33  1.01  1.63  2.07  0.57  0.44  -0.02  0.03  1.63  3.56  0.06  0.89  0.25  1.13  2.23  1.15  1.19  0.61  1.21  0.60  0.31  0.62  0.00  2.41  0.07  1.48  0.56  0.31  1.16  0.55  0.93  0.31  0.70  3.43  -0.11  0.81  1.12  1.05  0.54  3.42  1.32  0.95  1.20  0.95  0.05  -0.02  1.33  0.13  0.73  1.18  1.48  -0.38  3.08  0.38  -0.08  1.67  1.17  -0.05  0.53  1.17  0.71 | GFAGDDAPRAVFPS  RVAPEEHPTLL  AGDDAPRAVFPS  AGPAGPSGPRGPAGIA  KSYELPDGQVITIG  GAAGPAGPSGPRGPAGIA  DIDIRKDLYAN  VAPEEHPTLL  AGPSGPRGPAGIA  NWDDMEKIWHH  GQKDSYVGDEAQSKRGILTL  LRVAPEEHPTL  RGDSGPAGPPGEQGML  ELPDGQVITIG  SGSAGKDGMSGLPGPSGPSGPRGR  LLPVLYPPVVEE  SGPGGPTGPSGM  KSYELPDGQVITIGNE  GPAGPSGPRGPAGIA  FAGDDAPRAVFPS  IIAPPERKYS  KSYELPDGQVITI  GSAGPRGPSGNIGMPGMTGPQ  GEQIDNL  LGEQIDNL  GVDNPGHPF  TIIDQNRDGIIS  GETGKPGEQGLSGEAGAPGPAGS  KDLLDPIISDR  TGPIGPPGSGGAPGDKGE  GEKLKGADPEDVIVA  NVPVYEGY  GSAGPRGPSGNIGMPGMTGPQGE  GADPEDVIVA  GTDGAPGKDGIRGM  DLAGRDLTDYLMK  DLAGRDLTDYLM  GQKDSYVGDEAQSKRGIL  NPPKYDKIEDM  LEPVLVDIS  LVPIVEPE  VIDQDKSGFIE  KSYELPDGQVI  GKDGMSGLPGPSGPSGPRGR  KDDLRDVLA  TGDGVNDAPALK  GSSGPGGERGPPGPMGPS  VLYPPVVEE  LPVLYPPVVEE  GWLDKNKDPLNDSVVQ  GIVLDSGDGVTH  LGETNPADSKPGSIR  APEEHPTLL  GFAGDDAPRAVFPSIVGRPRHQ  IIDQDKSDFVEEDELKLF  KDLLDPIISD  DFENEMATAA  GPSGSSGSAGKDGMSGLPGPSGPSGPRGR  SGPSGPAGF  GPSGPRGPAGIA  AGFAGDDAPRAVFPS  IIDQNRDGIIS  GFNPPDLDIM  RGEQGPGGPGGF  TEAPLNPKANREKMTQIM  AINDPFIDL  GLAGDKGPSGDSGPA  ELPDGQVITI  SNKLLPVLYPPVVEE  GERGEQGPGGPGGF  LDKNKDPLNDSVVQ  SGDSGSAGPAGPSGPRGPAGPHGPPGKDGR  LDKVLPVE  GSLEDQIIA  GLVPIVEPE  DLAGRDLTDY  MGPAGPPGPPGPPGPPGAPGGGFDM  AGPRGPSGNIGMPGMTGPQ  NVLSGGTTMYPGIADRM | | 2.13  2.09  1.56  1.33  1.30  1.16  1.12  1.08  1.06  0.92  0.88  0.85  0.84  0.82  0.74  0.67  0.67  0.65  0.64  0.62  0.59  0.58  0.58  0.58  0.58  0.58  0.58  0.55  0.54  0.52  0.52  0.52  0.52  0.50  0.50  0.48  0.46  0.45  0.44  0.44  0.43  0.43  0.42  0.42  0.42  0.42  0.41  0.41  0.41  0.40  0.37  0.36  0.36  0.35  0.35  0.34  0.34  0.34  0.33  0.33  0.33  0.32  0.32  0.31  0.31  0.30  0.30  0.29  0.29  0.28  0.28  0.28  0.27  0.27  0.27  0.26  0.26  0.26  0.25 | 0.29  0.36  0.97  0.16  -0.78  0.16  -1.24  -0.53  -0.23  0.75  -0.71  0.64  -1.16  -0.99  -0.23  0.24  -1.24  -0.17  -0.10  0.35  -0.59  -0.70  -1.75  1.96  2.57  1.13  0.70  -0.06  0.86  -1.23  -1.01  -1.70  -1.64  -1.29  -0.30  1.02  0.65  -0.35  -0.15  1.23  0.77  0.37  -1.38  -0.08  1.37  -0.52  -1.26  -0.43  0.30  1.26  1.28  -1.42  -0.23  -0.75  -1.21  2.00  0.64  -0.40  -1.77  -0.46  0.53  0.68  -0.75  -1.42  -0.82  0.83  -1.51  -0.93  0.58  -0.79  1.41  -1.07  0.84  -0.12  0.65  0.72  -0.40  -1.67  1.49 | -0.86  -0.09  -1.33  -0.17  -0.79  -0.51  3.65  -0.81  -0.12  -0.27  -0.30  1.15  -0.62  0.01  -1.12  -1.23  -0.74  -0.82  -0.37  -0.89  -0.62  -0.82  -0.73  -1.14  -0.40  -0.49  -0.99  0.55  -0.66  -0.47  -0.41  0.66  -0.40  -1.00  -0.21  -0.45  -1.04  0.31  -0.70  0.08  -0.75  -0.43  -1.04  -0.72  -0.14  -0.82  -1.27  -1.16  -0.60  -0.75  -0.35  -0.89  -1.12  -0.81  0.00  -1.28  0.24  -1.13  -0.41  0.22  -0.61  -0.86  -0.19  -0.18  1.20  -0.47  -1.25  0.00  -1.25  -0.73  -0.79  -0.49  1.03  0.40  -0.69  -0.69  -0.92  -0.66  -0.74 | 0.71  1.79  1.23  0.47  2.64  0.23  0.83  1.14  1.26  1.02  1.27  1.24  0.13  2.07  0.32  1.33  -0.49  1.35  0.91  0.70  3.43  2.76  0.14  0.44  -0.40  0.08  1.49  0.87  0.80  0.55  2.36  0.56  -0.38  3.10  0.68  0.11  0.28  0.85  1.79  1.15  3.27  0.88  1.39  0.47  3.20  0.20  0.41  0.55  0.86  1.15  1.87  0.00  2.02  0.14  -0.16  0.34  1.77  0.45  0.42  1.65  0.36  1.67  1.20  1.04  1.12  0.47  0.49  2.37  -0.07  1.36  1.18  1.20  0.72  2.06  2.90  0.74  0.10  0.07  -0.46 | IIAPPERKYS  GVDNPGHPF  VAPEEHPTL  VLYPPVVEE  GADPEDVIVA  VIDQDKSGFIE  AGDDAPRAVFPS  KSYELPDGQ  GERGEQGPGGPGGF  IIDQNRDGIIS  AGPAGPSGPRGPAG  GLPGPSGPSGPRGR  RGEQGPGGPGGF  GFAGDDAPRA  AINDPFIDL  KAGDSDGDGAIGVD  GFAGDDAPRAVFPS  LEDQLSELK  KILDPEAT  GKDGMSGLPGPSGPSGPRGR  AGDSDGDGAIGVD  FSGDEEFPDLS  SAPKIPDGEKV  GDSDGDGAIGVD  VDFDDIQKK  TGPIGPPGSGGAPGDKGE  GERGEQGPGGPGGFQ  GKDGMSGLPGPS  FDDLPISEQ  TNWDDMEK  KAPEPAPEPEPAPVEPE  RVAPEEHPTLL  AGPPGVDGQPGAK  KTIDDLEDE  SGAPGPVGPA  GPPGPPGPPGPPGAPGGGFDM  LNEDKLKDKINE  VLYPPVVE  SGPGGPTGPSGM  GFNPPDLDIM  KSYELPDGQVI  KSYELPDGQVITIG  VIDQDKSGF  VEDEFPDLS  SGDEEFPDLS  GEQIDNL  VEWTDEE  IGPPGSGGAPGDKGE  IVPGDIVE  LKAGDSDGDGAIGVDEWAVLVKA  FDTDGGGDIS  SKLEDEQSI  RGDSGPAGPPGEQGM  LVPIVEPE  GNKDVILPVPA  VDEDGSGTIDFE  LGEQIDNL  LEEISERLEEAGGATSAQ  GFPGIPGPGGEPGKQ  SYELPDGQ  STGEPISEEE  VIDQDKSGFIEEDELKLF  APSADAPMFVM  LGETNPADSKPGS  LKAGDSDGDGAIGVD  KVEDEFPDLS  LYPPVVEE  LRGDSGPAGPPGEQGM  FQLPDERGYH  LDKVLPVE  FAGDDAPRA  TGVDNPGHPF  VDEDGSGTID | 3.49  2.00  1.75  1.65  1.59  1.53  1.43  1.35  1.20  1.19  1.17  1.17  1.16  1.12  1.08  0.95  0.87  0.83  0.81  0.79  0.78  0.77  0.76  0.76  0.69  0.67  0.67  0.63  0.59  0.58  0.54  0.52  0.52  0.51  0.49  0.47  0.47  0.46  0.44  0.44  0.43  0.42  0.41  0.40  0.40  0.39  0.39  0.39  0.37  0.36  0.35  0.35  0.35  0.34  0.33  0.33  0.32  0.32  0.31  0.28  0.28  0.27  0.27  0.27  0.27  0.26  0.26  0.26  0.26  0.25  0.25  0.25  0.25 | -0.59  1.13  -0.18  -0.43  -1.29  0.37  0.97  -1.09  -0.79  0.68  -0.56  -0.81  -1.42  0.45  0.83  -0.30  0.29  1.36  0.59  -0.08  -0.82  -0.08  -0.04  -1.11  -0.03  -1.23  -1.17  -0.20  -0.76  -0.04  -0.49  0.36  -0.95  2.19  0.08  -0.82  0.26  -0.08  -1.24  -0.75  -1.38  -0.78  0.25  1.60  0.49  1.96  0.21  -1.31  -0.18  -0.98  -0.77  -1.10  -0.60  0.77  -0.65  -0.41  2.57  1.34  0.50  -0.51  -0.34  -1.61  -1.44  -0.95  -0.49  1.82  0.73  0.04  -0.53  0.84  0.49  1.15  0.65 | -0.62  -0.49  -0.07  -1.16  -1.00  -0.43  -1.33  -0.94  -0.73  -0.86  -0.48  -1.11  -0.18  -0.67  -0.47  0.65  -0.86  -1.20  -0.71  -0.72  1.33  -0.84  -0.14  1.73  -0.87  -0.47  -0.25  -0.85  0.11  -1.27  1.04  -0.09  0.72  -0.23  -0.41  -0.74  0.12  -0.85  -0.74  -0.19  -1.04  -0.79  -0.87  -0.60  -1.31  -1.14  -0.53  -0.65  -1.12  -1.16  1.68  0.89  -1.11  -0.75  1.18  2.27  -0.40  -1.22  -1.18  -1.00  -1.28  -0.47  -0.11  -0.44  0.04  0.02  -0.36  -0.62  0.30  1.03  -0.69  -0.67  1.49 | 3.43  0.08  1.04  0.55  3.10  0.88  1.23  -0.55  1.36  1.67  0.21  0.80  1.04  1.06  0.47  1.81  0.71  -0.22  0.96  0.47  1.27  0.53  0.10  1.67  1.81  0.55  0.84  -0.04  0.52  0.09  0.63  1.79  0.69  1.13  -0.11  0.52  -0.11  -0.38  -0.49  1.20  1.39  2.64  1.30  -0.05  0.83  0.44  0.92  0.64  -0.22  1.53  -0.25  0.76  -0.34  3.27  3.08  0.06  -0.40  -0.02  1.80  0.05  0.37  -0.33  0.86  0.69  1.11  0.56  -0.12  -0.80  2.26  0.72  1.15  1.06  0.08 |  |
| HCM | | | | | | | | | | | | | | | | |
| Neut |  | α | β | γ | Alc | |  | α | β | γ | Neut&Alc |  | α | β | γ |  |
| LEQQVDDLEGSLEQEKK  LQGEVEDLMVDVERANG  IITNWDDMEK  VQHELEEAEERADIAETQVNK  LEKSYELPDGQVIT  VETEKTEIQSALEEAEGTLEHEESKILR  LDDLQAEEDKVNT  LADWKQKYEEGQAELEGSLKEARS  VAPEEHPTL  LDDVIQTGVDNPGHPFIMT  LEDECSELKKDIDDLELT  ARIEELEEELEAERA  LTEEMASQDESVAK  IEELEEELEAERA  LDFENEMAT  VDDIIQTGVDNPGHPFIMT  LKAGDSDGDGAIGVDEWA  LVQVQGEVDDSVQEARNAEEKAKKA  ALEEAEGTLEHEESKLLR  IEELEEELEAERAAR  LKGTEDELDKYSEALKDAQEKLE  LESDLVQVQGEVDDSVQEARNAEEKAKK  LTENGEFGRQLEEKEA  VMVGMGQKDSYVGDEAQSKRG  LAEKDEEMEQIKRNSQR  LKAGDSDGDGAIGVDEWAVL  MEGDLNEMELQ  VAGDEESYEVFKD  ILEEECMFPK  LTKLEEAEKAADESERGMK  FTPDQMEDYREAFG  ADWKQKYEEGQAELEGSLKEARS  ARIEELEEELEAERAAR  LESDLVQVQGEVDDSVQEARNAEEKAKKA  VQGEVDDSVQEARNAEEKAKKA  IIDQNRDGIISKDDLRD  AAGPAGPSGPRGPAG  IDASERVGL  VASIDDKEELDATDAAIDILG  VAQWRSKYETDAIQRTEELEESKKK  LEEAEGTLEHEESKLLR  VQVQGEVDDSVQEARNAEEKAKKA  ISDLTEQLGETGKS  LTEQLGETGKS  LEDQLSELK  ADSVAELGEQIDNLQR  TEKTEIQSALEEAEGTLEHEESKILRVQ  LEDQLSEIKAKSDENARQ  LEKTIDDLEDELYAQK  LEKSYELPDGQVITIGNERFRCPET  LKGADPEDVIVAA  FSGDEEFPDLS  FTVDDIIQTGVDNPGHPFIMT  IDDLEDELYAQK  LAQESIMDLENDKQQSDEKLKKKDFETSQ  LKKKMEGDLNEMELQ  FVIDQDKSGFIEEDELK  IVLDSGDGVTHNVPVYEGY  LDDAVRAAEDLKEQAAM  VETEKTEIQSALEEAEGTLEHEESK  VIDQDKSGFIEEDELKLF  VAEQELIDASERVGL  VLADWKQKYEEGQAELEGSLKEARS  LQEAEEQIEAVNSK  VALDFENEMAT  LDDAVRAAEDLKEQ  LEKEKSEYKMEIDDLSSNMEAVSKAKGN  VLSGGTTMYPG  IEELEEELEAERAARAK  LKAGDSDGDGKIGVDEFGAM  LEKEKSEYKMEIDDLSSNMEA  SAPKIPDGEKVDFDDIQKKRQNKD  IVLDSGDGVTHNVPVYEGYALPHA  LVDASERVGL  MGQKDSYVGDEAQSKRG  LEEISERLEEAGGATSAQ  VGMGQKDSYVGDEAQSKRG  LKGGDDLDPAYV  MEIDDLSSNMEA  LREQFEEEQEAKAE  ILGNPSDEDMNSKRVDFEG  IEELEEELEAERAARAKVEKQRAD  VMDLENDKQQSDEKLKKKDFETSQL  LADWKQKYEEGQAELEGS  LRGDSGPAGPPGEQGM  LDDVIQTGVDNPGHPF  VQHELEEAEERADIAETQ  LQEAEEQIEAVNSKCAS  LGAPGPSGAPGPVGPAGKTGDRGETGPAG  VIDQDKSGFIEEDELK  LKAGDSDGDGAIGVDEWAV | 2.38  2.28  2.23  1.46  1.27  1.00  0.94  0.74  0.72  0.72  0.71  0.71  0.68  0.67  0.66  0.65  0.65  0.63  0.61  0.60  0.58  0.56  0.54  0.53  0.53  0.53  0.53  0.52  0.51  0.51  0.51  0.50  0.49  0.48  0.47  0.46  0.46  0.44  0.43  0.43  0.43  0.41  0.40  0.39  0.38  0.38  0.36  0.35  0.35  0.35  0.35  0.35  0.33  0.33  0.33  0.33  0.32  0.32  0.32  0.32  0.32  0.31  0.31  0.31  0.31  0.31  0.31  0.31  0.30  0.30  0.29  0.29  0.29  0.28  0.28  0.28  0.28  0.28  0.27  0.27  0.26  0.26  0.26  0.26  0.26  0.25  0.24  0.23  0.23  0.23  0.22 | 2.18  1.38  0.75  2.21  -0.55  2.29  0.55  0.70  -0.18  0.83  1.84  -0.65  1.01  0.22  0.10  0.89  0.26  2.64  1.83  -0.70  2.96  2.83  0.10  -0.69  1.24  -0.38  -0.76  0.01  -0.43  1.53  0.24  0.32  -1.60  2.76  2.82  -0.51  -0.56  0.02  0.00  0.86  1.91  2.26  2.57  1.54  1.36  2.85  1.40  1.54  1.51  0.47  -1.53  -0.08  0.54  0.45  1.01  -0.04  -1.60  0.37  2.43  1.33  -1.61  0.21  0.96  1.65  0.13  2.93  0.79  -0.22  -0.32  -1.32  0.33  -0.05  0.51  0.75  -0.62  1.34  -1.22  -0.18  0.23  0.07  -0.76  -0.02  0.66  -0.29  0.04  1.96  1.07  1.80  -0.15  -1.23  0.26 | -0.87  -0.45  -1.28  -0.41  -0.43  -0.80  -1.02  -0.08  -0.07  0.33  -0.42  -0.93  -0.17  0.05  1.04  0.34  -0.18  1.54  -0.16  0.82  0.22  0.55  0.33  -0.91  -0.99  -0.26  -1.27  -1.11  -0.67  -0.55  -1.18  -0.47  -0.13  0.38  1.33  -0.56  -0.75  2.31  -0.74  -0.28  -0.37  2.04  -0.34  0.07  -1.20  0.23  -0.80  0.10  -0.63  -1.10  -0.19  -0.84  0.70  0.14  -1.19  -0.30  -0.95  0.67  1.34  -1.19  -0.47  0.78  -0.57  -1.27  1.18  1.36  -0.30  -0.94  1.45  -0.23  -1.03  -0.25  0.31  1.40  0.01  -1.22  -0.62  -0.37  -0.61  2.42  -0.19  0.73  -0.93  0.27  -0.62  0.23  -0.85  -1.25  0.44  -0.56  -0.72 | 1.26  0.55  2.95  0.64  0.82  0.25  1.17  0.43  1.04  -0.02  0.82  0.54  0.11  1.05  0.59  0.07  0.56  2.42  0.57  0.31  0.47  2.23  0.33  2.46  1.67  1.46  -0.11  0.93  0.38  1.15  0.00  0.89  0.76  1.88  1.45  0.70  0.55  1.12  1.40  1.20  0.55  1.38  1.01  0.25  -0.22  1.13  0.08  1.33  0.12  0.31  2.78  0.53  0.34  1.00  3.56  -0.07  1.26  0.60  0.27  0.70  -0.33  0.07  0.37  0.25  1.63  1.24  0.66  0.44  0.45  1.39  1.48  2.07  0.05  0.41  0.18  -0.02  0.92  0.44  -0.60  1.03  1.63  0.88  1.21  1.19  -0.80  0.85  0.68  0.54  0.86  0.62  1.09 | AGDDAPRAVFPS  GPAGPSGPRGPAGIA  AGPAGPSGPRGPAGIA  VAPEEHPTLL  GFAGDDAPRAVFPS  GAAGPAGPSGPRGPAGIA  RVAPEEHPTLL  ELPDGQVITIG  KSYELPDGQVITIG  LLPVLYPPVVEE  LGEQIDNL  RGDSGPAGPPGEQGML  GSAGPRGPSGNIGMPGMTGPQ  TIIDQNRDGIIS  GEKLKGADPEDVIVA  SGSAGKDGMSGLPGPSGPSGPRGR  SGPGGPTGPSGM  GEQIDNL  ELPDGQVI  KSYELPDGQVITI  TGPIGPPGSGGAPGDKGE  FAGDDAPRAVFPS  SNKLLPVLYPPVVEE  GSAGPRGPSGNIGMPGMTGPQGE  NPPKYDKIEDM  GFNPPDLDIM  LRVAPEEHPTL  GVDNPGHPF  GWLDKNKDPLNDSVVQ  KDLLDPIISDR  KSYELPDGQVI  GSSGPGGERGPPGPMGPS  ELPDGQVITI  AGFAGDDAPRAVFPS  EASGPINF  SAGKDGMSGLPGPSGPSGPRGR  AGPRGPSGNIGMPGMTGPQ  SGPSGPAGF  KDDLRDVLA  KDLLDPIISD  KLLPVLYPPVVEE  LTDRENQSVL  DLAGRDLTDYLM  GKDGMSGLPGPSGPSGPRGR  MGPAGPPGPPGPPGPPGAPGGGFDM  GSLEDQIIA  GETGKPGEQGLSGEAGAPGPAGS  EASGPINFT  FQLPDERGYHIF  DLAGRDLTDY  LLPVLYPPVVE  SPNKGTLDDYVE  KKAEPAPEPVAVPAPK  GDRGVPGDQGLAGPA  GERGEQGPGGPGGF  LPVLYPPVVEE  GAPGGGWVFPAQPL  EIVPGDIVE  GADPEDVIVA  TIVNSPNKGTLDDYVE  TIIDQNRDGIISK  FKILDPEAT  GDDAPRAVFPS  KAPEPAPEPEPAPVEPE  FDDLPISEQ  FSGDEEFPDL  GVDNPGHPFIM  DIDYKPLPF  GDQGPAGSAGPAGPRGPSGS  VLYPPVVEE  NKLLPVLYPPVVEE  EKTIDDLEDE  GPPGPPGPPGPPGAPGGGFDM  GSAGPRGPSGNIGMPGMTGPQGEAGRE  LEPVLVDIS  LDKVLPVE  DFVEEDELKLF  TGDGVNDAPALK  IIDQNRDGIIS | | 3.13  2.64  1.90  1.87  1.75  1.62  1.53  1.42  1.34  1.21  1.09  1.03  0.94  0.90  0.85  0.84  0.76  0.74  0.70  0.67  0.67  0.67  0.66  0.65  0.63  0.63  0.61  0.61  0.57  0.56  0.56  0.56  0.52  0.49  0.48  0.47  0.46  0.42  0.41  0.40  0.38  0.38  0.37  0.36  0.36  0.34  0.34  0.33  0.33  0.32  0.32  0.32  0.32  0.32  0.31  0.31  0.31  0.31  0.30  0.30  0.30  0.29  0.28  0.28  0.28  0.28  0.28  0.28  0.28  0.27  0.26  0.26  0.26  0.25  0.25  0.25  0.25  0.25  0.24 | 0.97  -0.10  0.16  -0.53  0.29  0.16  0.36  -0.99  -0.78  0.24  2.57  -1.16  -1.75  0.70  -1.01  -0.23  -1.24  1.96  -1.92  -0.70  -1.23  0.35  0.58  -1.64  -0.15  -0.75  0.64  1.13  1.26  0.86  -1.38  -1.26  -0.93  0.53  -0.16  0.00  -1.67  -1.77  1.37  2.00  0.93  -0.20  0.65  -0.08  -0.40  -0.12  -0.06  -0.41  0.31  0.72  0.84  0.15  0.98  0.19  -0.79  0.30  -1.38  0.11  -1.29  -0.61  0.24  0.53  0.95  -0.49  -0.76  0.15  0.59  -0.32  -1.07  -0.43  0.84  2.02  -0.82  -0.89  1.23  0.84  0.11  -0.52  0.68 | -1.33  -0.37  -0.17  -0.81  -0.86  -0.51  -0.09  0.01  -0.79  -1.23  -0.40  -0.62  -0.73  -0.99  -0.41  -1.12  -0.74  -1.14  -0.85  -0.82  -0.47  -0.89  -1.25  -0.40  -0.70  -0.19  1.15  -0.49  -0.75  -0.66  -1.04  -1.27  0.00  -0.61  2.15  -1.06  -0.66  -0.41  -0.14  -1.28  -0.85  -0.79  -1.04  -0.72  -0.92  0.40  0.55  2.09  0.44  -0.69  -0.65  0.94  -0.59  0.04  -0.73  -0.60  -0.73  -0.90  -1.00  0.95  -1.08  -1.23  -1.02  1.04  0.11  -0.69  -0.16  -0.54  -0.39  -1.16  -1.30  -0.91  -0.74  -0.35  0.08  1.03  0.43  -0.82  -0.86 | 1.23  0.91  0.47  1.14  0.71  0.23  1.79  2.07  2.64  1.33  -0.40  0.13  0.14  1.49  2.36  0.32  -0.49  0.44  0.68  2.76  0.55  0.70  -0.07  -0.38  1.79  1.20  1.24  0.08  1.15  0.80  1.39  0.41  2.37  0.36  0.27  0.51  0.07  0.42  3.20  0.34  0.65  2.58  0.28  0.47  0.10  2.06  0.87  0.04  0.74  0.74  0.91  -0.21  1.50  1.39  1.36  0.86  -0.12  -0.34  3.10  0.65  1.05  1.37  1.63  0.63  0.52  0.47  1.86  1.25  -0.87  0.55  0.10  0.41  0.52  -0.27  1.15  0.72  1.50  0.20  1.67 | VAPEEHPTL  VLYPPVVEE  IIDQNRDGIIS  ELPDGQVIT  VIDQDKSGFIE  IIAPPERKYS  LEDQLSELK  GVDNPGHPF  GADPEDVIVA  AAGPAGPSGPRGPAG  AGDDAPRAVFPS  LDKNKDPLNDSVVQ  FAGDDAPRA  ELPDGQVITI  KSYELPDGQVITIG  GFNPPDLDIM  ELPDGQVI  NPPKYDKIEDM  KSYELPDGQVITI  KSYELPDGQVIT  ELPDGQVITIG  AGPAGPSGPRGPAG  GERGEQGPGGPGGF  SGPGGPTGPSGM  KILDPEAT  GKDGMSGLPGPSGPSGPRGR  FSGDEEFPDLS  GSSGSAGKDGMSGLPGPSGPSGPRGR  EASGPINF  KSYELPDGQVI  KSYELPDGQ  FQLPDERGYH  LVPIVEPE  SGSAGKDGMSGLPGPSGPSGPRGR  RGEQGPGGPGGF  TGPIGPPGSGGAPGDKGE  LRGDSGPAGPPGEQGM  KAPEPAPEPEPAPVEPE  GPPGPPGPPGPPGAPGGGFDM  IVPGDIVE  GSSGPGGERGPPGPMGPSG  GLPGPSGPSGPRGR  GEKLKGADPEDVIVA  VLYPPVVE  GSLEDQIIA  SAGKDGMSGLPGPSGPSGPRGR  IIDQDKSDFVEEDELK  LLPVLYPPVVEE  LGETNPADSKPGSIR  FDDLPISEQ  LTDRENQSVL  IGPPGSGGAPGDKGE  AINDPFIDL  SAPKIPDGEKV  LNEDKLKDKINE  LGEQIDNL  MGPAGPPGPPGPPGPPGAPGGGFDM  IIDQNRDGIISK  GSSGPGGERGPPGPM  SPNKGTLDDYVE | 1.91  1.75  1.45  1.14  1.13  1.11  1.07  1.06  1.00  0.95  0.75  0.71  0.68  0.67  0.67  0.63  0.60  0.60  0.60  0.58  0.57  0.56  0.55  0.55  0.54  0.54  0.54  0.54  0.53  0.51  0.50  0.50  0.49  0.48  0.46  0.46  0.45  0.44  0.44  0.43  0.43  0.41  0.40  0.39  0.38  0.38  0.38  0.38  0.38  0.37  0.35  0.35  0.34  0.34  0.34  0.34  0.33  0.33  0.31  0.31 | -0.181  -0.43  0.68  -1.76  0.37  -0.59  1.36  1.13  -1.29  -0.56  0.97  1.41  0.49  -0.93  -0.78  -0.75  -1.92  -0.15  -0.70  -1.28  -0.99  -0.56  -0.79  -1.24  0.59  -0.08  -0.08  -0.18  -0.16  -1.38  -1.09  -0.53  0.77  -0.23  -1.42  -1.23  0.04  -0.49  -0.82  -0.18  -1.27  -0.81  -1.01  -0.08  -0.12  0.00  -1.22  0.24  -1.42  -0.76  -0.20  -1.31  0.83  -0.04  0.26  2.57  -0.40  0.17  -1.10  0.15 | -0.069  -1.16  -0.86  -0.72  -0.43  -0.62  -1.20  -0.49  -1.00  -0.75  -1.33  -0.79  -0.69  0.00  -0.79  -0.19  -0.85  -0.70  -0.82  -1.17  0.01  -0.48  -0.73  -0.74  -0.71  -0.72  -0.84  -1.11  2.15  -1.04  -0.94  0.30  -0.75  -1.12  -0.18  -0.47  -0.62  1.04  -0.74  -1.12  -1.29  -1.11  -0.41  -0.85  0.40  -1.06  -0.06  -1.23  -0.89  0.11  -0.79  -0.65  -0.47  -0.14  0.12  -0.40  -0.92  -1.18  -1.19  0.94 | 1.042  0.55  1.67  1.69  0.88  3.43  -0.22  0.08  3.10  0.55  1.23  1.18  1.15  2.37  2.64  1.20  0.68  1.79  2.76  1.13  2.07  0.21  1.36  -0.49  0.96  0.47  0.53  0.10  0.27  1.39  -0.55  2.26  3.27  0.32  1.04  0.55  -0.80  0.63  0.52  -0.22  0.41  0.80  2.36  -0.38  2.06  0.51  0.20  1.33  0.00  0.52  2.58  0.64  0.47  0.10  -0.11  -0.40  0.10  0.90  0.05  -0.21 |  |

**Table S3.** All peptide data from proteomics analysis including relative intensities, weighted length, weighted charge, weighted molecular weight, weighted AA distribution, and in sample ranking (by intensity) (Table is appended separately as .xlsx file).

**Table S4.** Sequences, relative intensity, and predicted free radical scavenging and (FRS) metal chelating activity (MCA) scores for the 100 peptides with highest relative intensity from enzymatically derived FPH from minced cod frame (MCF) and heated cod meat (HCM).

| **MCF** | | | | | | | | | | | |
| --- | --- | --- | --- | --- | --- | --- | --- | --- | --- | --- | --- |
| **Neut.** | **Rel. Int. (%)** | **Score** | | Alc | Rel. Int. (%) | Score | | Neut&Alc | Rel. Int. (%) | Score | |
|  |  | **FRS** | **MCA** |  |  | FRS | MCA |  |  | FRS | MCA |
| LQGEVEDLMVDVERANG  LEQQVDDLEGSLEQEKK  IITNWDDMEK  VQHELEEAEERADIAETQVNK  LTKLEEAEKAADESERGMK  LEDQLSEIKAKSDENARQ  LEKSYELPDGQVIT  VAPEEHPTL  IIDQNRDGIISKDDLRD  LDDLQAEEDKVNT  LEKTIDDLEDELYAQK  LKGTEDELDKYSEALKDAQEKLE  LTEEMASQDESVAK  LKGADPEDVIVAA  LADWKQKYEEGQAELEGSLKEARS  SKYETDAIQRTEELEESKKK  LKAGDSDGDGAIGVDEWAV  LTDAETKAF  LKAGDSDGDGAIGVDEWAVLVKA  VDDIIQTGVDNPGHPFIMT  VIDQDKSGFIEEDELKLF  VMVGMGQKDSYVGDEAQSKRG  LDFENEMAT  IDDLEDELYAQK  VQGEVDDSVQEARNAEEKAKKA  ARIEELEEELEAERA  LVQVQGEVDDSVQEARNAEEKAKKA  ARIEELEEELEAERAAR  VASIDDKEELDATDAAIDILG  FVIDQDKSGFIEEDELK  LDDAVRAAEDLKEQAAM  VAGDEESYEVFKD  LEKEKSEYKMEIDDLSSNMEAVSKAKGN  ILDPEATGSIKKEF  LEDECSELKKDIDDLELT  LTENGEFGRQLEEKEA  ISDLTEQLGETGKS  LVEEELDRAQER  SAPKIPDGEKVDFDDIQKKRQNKD  ALEEAEGTLEHEESKLLR  VLSGGTTMYPG  LDDVIQTGVDNPGHPFIMT  ILEEECMFPKASDATFKAKL  VALDFENEMAT  LAQESIMDLENDKQQSDEKLKKKDFETSQ  ISEELDHALNDMTSI  ADWKQKYEEGQAELEGSLKEARS  LNVKNEELEAMVKE  ADSVAELGEQIDNLQR  LESDLVQVQGEVDDSVQEARNAEEKAKK  IGMESAGIHET  VRDLESEVDNEQRRGAEA  GPSGAPGPVGPAGKTGDRGETGPAG  VMDLENDKQQSDEKLKKKDFETSQL  IVLDSGDGVTHNVPVYEGY  LEKSYELPDGQVITIGNERFRCPET  VIDQDKSGFIEEDELK  FTPDQMEDYREAFG  LAQESIMDLENDKQQSDEKLKKKDFETSQL  VAEQELIDASERVGL  LEKEKSEYKMEIDDLSSNMEA  LFQPSF  LKAGDSDGDGAIGVDEWA  LDDAIRAADDLKEQAAM  DWKQKYEEGQAELEGSLKEARS  AAGPAGPSGPRGPAG  LDDAVRAAEDLKEQA  IEELEEELEAERAAR  VETEKTEIQSALEEAEGTLEHEESK  IIAPPERKYS  MEGDLNEMELQ  LKREEADAKKKMEEDAKKKSA  IDASERVGL  IEELEEELEAERA  LQEAEEQIEAVNSKCAS  LVIIESDLERTEERAE  IGMESAGIHETAYNS  VDDIIQTGVDNPGHPF  VAQWRSKYETDAIQRTEELEESKKK  LVEEELDRAQERL  MEIDDLSSNMEAVSKAKGN  LEEISERLEEAGGATSAQ  MAEELKKEQDTSSHLERMKKN  LEKSESIDDMIPAQK  AMKDEEKMELQEIQ  LDKNKDPLNDSVVQ  GEVDDSVQEARNAEEKAKKA  ADGNVGPAGPAGPLG  VIIESDLERTEERAE  ILEEECMFPK  ILADADCAAA  LAEKDEEMEQIKRNSQR  LSKIEDEQSLGAQ  FEQSQIQEYKEAFT  FSGDEEFPDLS  LKETTERLEDEEEINAE  ILDPEATGSIKKE | 1.94  1.92  1.78  1.39  1.17  1.00  0.92  0.90  0.86  0.81  0.78  0.77  0.76  0.76  0.71  0.69  0.69  0.65  0.61  0.59  0.57  0.54  0.54  0.54  0.53  0.53  0.52  0.52  0.52  0.51  0.49  0.48  0.47  0.47  0.45  0.43  0.42  0.41  0.41  0.39  0.39  0.38  0.37  0.36  0.35  0.35  0.35  0.34  0.33  0.33  0.32  0.32  0.32  0.32  0.32  0.32  0.31  0.31  0.31  0.31  0.31  0.30  0.30  0.29  0.29  0.29  0.29  0.28  0.28  0.28  0.27  0.27  0.27  0.27  0.26  0.26  0.26  0.26  0.26  0.25  0.25  0.25  0.25  0.24  0.24  0.24  0.23  0.23  0.23  0.23  0.22  0.22  0.22  0.22  0.21  0.21  0.21 | 0.33  0.38  0.38  0.33  0.29  0.23  0.48  0.51  0.30  0.34  0.35  0.34  0.31  0.38  0.30  0.40  0.35  0.33  0.36  0.42  0.36  0.46  0.37  0.40  0.39  0.36  0.34  0.39  0.35  0.38  0.34  0.51  0.22  0.35  0.26  0.40  0.40  0.38  0.32  0.47  0.45  0.41  0.32  0.39  0.25  0.44  0.32  0.36  0.41  0.23  0.41  0.30  0.42  0.27  0.57  0.34  0.37  0.41  0.25  0.28  0.29  0.42  0.35  0.29  0.35  0.49  0.33  0.37  0.23  0.40  0.38  0.29  0.31  0.36  0.33  0.35  0.44  0.38  0.27  0.36  0.35  0.38  0.36  0.33  0.37  0.29  0.34  0.50  0.34  0.45  0.29  0.36  0.28  0.43  0.43  0.35  0.37 | 0.17  0.19  0.19  0.15  0.17  0.17  0.23  0.25  0.22  0.23  0.22  0.16  0.21  0.19  0.15  0.14  0.18  0.24  0.13  0.20  0.19  0.13  0.25  0.27  0.14  0.20  0.11  0.19  0.21  0.20  0.19  0.21  0.12  0.23  0.18  0.21  0.22  0.25  0.18  0.22  0.20  0.21  0.14  0.22  0.16  0.26  0.17  0.20  0.23  0.11  0.21  0.21  0.18  0.16  0.23  0.13  0.22  0.23  0.18  0.20  0.12  0.28  0.19  0.20  0.17  0.22  0.21  0.21  0.20  0.23  0.27  0.11  0.21  0.20  0.18  0.19  0.20  0.21  0.15  0.26  0.21  0.19  0.16  0.21  0.24  0.27  0.16  0.25  0.19  0.23  0.22  0.21  0.22  0.21  0.23  0.19  0.22 | GFAGDDAPRAVFPS  RVAPEEHPTLL  AGDDAPRAVFPS  AGPAGPSGPRGPAGIA  FLGM  KSYELPDGQVITIG  GAAGPAGPSGPRGPAGIA  DIDIRKDLYAN  VAPEEHPTLL  AGPSGPRGPAGIA  NWDDMEKIWHH  GQKDSYVGDEAQSKRGILTL  NWDDME  LRVAPEEHPTL  RGDSGPAGPPGEQGML  ELPDGQVITIG  SGSAGKDGMSGLPGPSGPSGPRGR  LDLL  LLPVLYPPVVEE  SGPGGPTGPSGM  KSYELPDGQVITIGNE  GPAGPSGPRGPAGIA  VGPF  FAGDDAPRAVFPS  IIAPPERKYS  FETF  KSYELPDGQVITI  GSAGPRGPSGNIGMPGMTGPQ  GEQIDNL  LGEQIDNL  GVDNPGHPF  TIIDQNRDGIIS  GETGKPGEQGLSGEAGAPGPAGS  KDLLDPIISDR  TGPIGPPGSGGAPGDKGE  GEKLKGADPEDVIVA  NVPVYEGY  GSAGPRGPSGNIGMPGMTGPQGE  GADPEDVIVA  GTDGAPGKDGIRGM  LLME  DLAGRDLTDYLMK  VFDL  VFLD  DLAGRDLTDYLM  LLEM  VAVL  GQKDSYVGDEAQSKRGIL  NPPKYDKIEDM  LEPVLVDIS  LVPIVEPE  VIDQDKSGFIE  KSYELPDGQVI  LDFENE  GKDGMSGLPGPSGPSGPRGR  KDDLRDVLA  TGDGVNDAPALK  GSSGPGGERGPPGPMGPS  VLYPPVVEE  LPVLYPPVVEE  GWLDKNKDPLNDSVVQ  LFDF  GIVLDSGDGVTH  LGETNPADSKPGSIR  APEEHPTLL  GFAGDDAPRAVFPSIVGRPRHQ  IIDQDKSDFVEEDELKLF  KDLLDPIISD  DFENEMATAA  GPSGSSGSAGKDGMSGLPGPSGPSGPRGR  SGPSGPAGF  GPSGPRGPAGIA  AGFAGDDAPRAVFPS  IIDQNRDGIIS  GFNPPDLDIM  RGEQGPGGPGGF  TEAPLNPKANREKMTQIM  AINDPFIDL  GLAGDKGPSGDSGPA  ELPDGQVITI  FLPM  SNKLLPVLYPPVVEE  GERGEQGPGGPGGF  LDKNKDPLNDSVVQ  SGDSGSAGPAGPSGPRGPAGPHGPPGKDGR  TLDDL  LDKVLPVE  GSLEDQIIA  GLVPIVEPE  DLAGRDLTDY  LDFE  LLGW  MGPAGPPGPPGPPGPPGAPGGGFDM  AGPRGPSGNIGMPGMTGPQ  NVLSGGTTMYPGIADRM | 2.30  2.13  2.09  1.56  1.34  1.33  1.30  1.16  1.12  1.08  1.06  0.92  0.88  0.85  0.84  0.74  0.67  0.67  0.65  0.64  0.62  0.59  0.58  0.58  0.58  0.58  0.58  0.58  0.54  0.52  0.52  0.52  0.52  0.50  0.50  0.48  0.46  0.45  0.44  0.44  0.43  0.43  0.42  0.42  0.42  0.42  0.41  0.41  0.41  0.40  0.37  0.36  0.36  0.35  0.35  0.34  0.34  0.34  0.33  0.33  0.33  0.32  0.31  0.31  0.30  0.30  0.29  0.29  0.28  0.28  0.28  0.27  0.27  0.27  0.26  0.26  0.25  0.25  0.24  0.24  0.24  0.23  0.23  0.23  0.22  0.22  0.21  0.21  0.21  0.21  0.20  0.20  0.20  0.20  0.19 | 0.51  0.51  0.41  0.51  0.43  0.35  0.49  0.27  0.56  0.47  0.34  0.30  0.40  0.45  0.51  0.31  0.55  0.35  0.64  0.50  0.33  0.49  0.47  0.50  0.40  0.40  0.44  0.48  0.34  0.35  0.56  0.29  0.37  0.33  0.47  0.38  0.50  0.44  0.32  0.35  0.38  0.35  0.35  0.36  0.35  0.39  0.33  0.34  0.37  0.29  0.38  0.30  0.45  0.37  0.49  0.29  0.36  0.61  0.52  0.63  0.28  0.38  0.38  0.34  0.54  0.43  0.33  0.33  0.33  0.46  0.44  0.45  0.48  0.29  0.39  0.53  0.36  0.38  0.41  0.33  0.46  0.53  0.52  0.29  0.54  0.32  0.35  0.30  0.40  0.35  0.36  0.48  0.68  0.47  0.55 | 0.25  0.26  0.24  0.21  0.28  0.21  0.20  0.17  0.26  0.20  0.20  0.18  0.21  0.25  0.29  0.20  0.25  0.28  0.21  0.23  0.17  0.21  0.27  0.25  0.23  0.25  0.23  0.23  0.22  0.22  0.28  0.20  0.23  0.25  0.22  0.17  0.19  0.23  0.20  0.21  0.28  0.21  0.25  0.25  0.21  0.27  0.23  0.18  0.18  0.22  0.22  0.19  0.24  0.27  0.25  0.21  0.26  0.23  0.21  0.21  0.23  0.27  0.19  0.20  0.27  0.26  0.21  0.26  0.24  0.19  0.28  0.21  0.23  0.19  0.25  0.23  0.18  0.24  0.22  0.20  0.31  0.21  0.23  0.27  0.23  0.23  0.23  0.22  0.21  0.19  0.27  0.27  0.31  0.22  0.17 | IIAPPERKYS  GVDNPGHPF  LDLL  VGPF  VAPEEHPTL  VLYPPVVEE  GADPEDVIVA  VIDQDKSGFIE  AGDDAPRAVFPS  KSYELPDGQ  VAVL  LFPE  GLVL  LLEM  GERGEQGPGGPGGF  LGVL  LDFENE  IIDQNRDGIIS  AGPAGPSGPRGPAG  LLLS  GLPGPSGPSGPRGR  FETF  RGEQGPGGPGGF  VLTL  GFAGDDAPRA  VWLG  AINDPFIDL  NWDDM  KAGDSDGDGAIGVD  GFAGDDAPRAVFPS  LEDQLSELK  KILDPEAT  GKDGMSGLPGPSGPSGPRGR  AGDSDGDGAIGVD  LDF  FSGDEEFPDLS  FLGM  NWDDME  SAPKIPDGEKV  GDSDGDGAIGVD  VDFDDIQKK  TGPIGPPGSGGAPGDKGE  LFQPS  GERGEQGPGGPGGFQ  GKDGMSGLPGPS  FDDLPISEQ  TNWDDMEK  KAPEPAPEPEPAPVEPE  RVAPEEHPTLL  AGPPGVDGQPGAK  VLTLG  LLWE  KTIDDLEDE  SGAPGPVGPA  GPPGPPGPPGPPGAPGGGFDM  FVLD  LNEDKLKDKINE  VLYPPVVE  SGPGGPTGPSGM  GFNPPDLDIM  KSYELPDGQVI  KSYELPDGQVITIG  VIDQDKSGF  VEDEFPDLS  SGDEEFPDLS  LAEAP  GEQIDNL  VEWTDEE  IGPPGSGGAPGDKGE  VLDL  IVPGDIVE  LKAGDSDGDGAIGVDEWAVLVKA  FDTDGGGDIS  SKLEDEQSI  RGDSGPAGPPGEQGM  LVPIVEPE  GNKDVILPVPA  VDEDGSGTIDFE  LGEQIDNL  LEEISERLEEAGGATSAQ  GFPGIPGPGGEPGKQ  SYELPDGQ  VALW  STGEPISEEE  PTFE  VIDQDKSGFIEEDELKLF  APSADAPMFVM  VTVL  LGETNPADSKPGS  LKAGDSDGDGAIGVD  KVEDEFPDLS  LYPPVVEE  LLEL  LRGDSGPAGPPGEQGM  FQLPDERGYH  LDKVLPVE  FAGDDAPRA  FEEF  TGVDNPGHPF  VDEDGSGTID | 3.49  2.00  1.75  1.65  1.59  1.53  1.43  1.35  1.20  1.19  1.17  1.17  1.16  1.12  1.08  0.95  0.87  0.83  0.81  0.79  0.78  0.77  0.76  0.76  0.69  0.67  0.67  0.63  0.59  0.58  0.54  0.52  0.52  0.51  0.49  0.47  0.47  0.46  0.44  0.44  0.43  0.42  0.41  0.40  0.40  0.39  0.39  0.39  0.37  0.36  0.35  0.35  0.35  0.34  0.33  0.33  0.32  0.32  0.31  0.28  0.28  0.27  0.27  0.27  0.27  0.26  0.26  0.26  0.26  0.25  0.25  0.25  0.25  0.25  0.24  0.24  0.24  0.24  0.24  0.24  0.23  0.23  0.23  0.22  0.21  0.21  0.21  0.20  0.20  0.20  0.20  0.20  0.19  0.19  0.19  0.19  0.19  0.19  0.18  0.18 | 0.40  0.56  0.35  0.47  0.51  0.52  0.32  0.30  0.41  0.44  0.33  0.44  0.37  0.39  0.52  0.38  0.37  0.29  0.49  0.34  0.49  0.40  0.53  0.35  0.38  0.49  0.38  0.43  0.32  0.51  0.34  0.33  0.49  0.33  0.37  0.43  0.43  0.40  0.35  0.37  0.32  0.47  0.40  0.51  0.46  0.37  0.39  0.52  0.51  0.44  0.35  0.46  0.28  0.45  0.56  0.36  0.25  0.52  0.50  0.39  0.45  0.35  0.30  0.40  0.39  0.40  0.34  0.44  0.51  0.34  0.36  0.36  0.40  0.28  0.51  0.38  0.34  0.37  0.35  0.38  0.55  0.44  0.43  0.36  0.39  0.36  0.43  0.33  0.36  0.33  0.39  0.41  0.37  0.52  0.44  0.35  0.37  0.42  0.55  0.36 | 0.23  0.28  0.28  0.27  0.25  0.21  0.20  0.19  0.24  0.23  0.23  0.30  0.24  0.27  0.23  0.25  0.27  0.19  0.22  0.29  0.22  0.25  0.23  0.26  0.21  0.24  0.24  0.21  0.23  0.25  0.25  0.26  0.25  0.24  0.26  0.23  0.28  0.21  0.21  0.23  0.25  0.22  0.28  0.24  0.23  0.30  0.21  0.26  0.26  0.19  0.24  0.26  0.21  0.24  0.26  0.25  0.20  0.21  0.23  0.25  0.24  0.21  0.21  0.25  0.26  0.26  0.22  0.20  0.22  0.26  0.21  0.13  0.25  0.23  0.27  0.22  0.19  0.24  0.22  0.19  0.27  0.27  0.23  0.24  0.27  0.19  0.23  0.23  0.23  0.20  0.24  0.21  0.28  0.27  0.21  0.23  0.22  0.25  0.28  0.26 |
| HCM | | | | | | | | | | | |
| Neut. | Rel. Int. (%) | FRS | MCA | Alc. | Rel. Int. (%) | FRS | MCA | Netu&Alc | Rel. Int. (%) | FRS | MCA |
| LEQQVDDLEGSLEQEKK  LQGEVEDLMVDVERANG  IITNWDDMEK  VQHELEEAEERADIAETQVNK  LEKSYELPDGQVIT  VETEKTEIQSALEEAEGTLEHEESKILR  LDDLQAEEDKVNT  LADWKQKYEEGQAELEGSLKEARS  VAPEEHPTL  LFQPSF  LDDVIQTGVDNPGHPFIMT  LEDECSELKKDIDDLELT  ARIEELEEELEAERA  LTEEMASQDESVAK  FLPM  IEELEEELEAERA  LDFENEMAT  VDDIIQTGVDNPGHPFIMT  LKAGDSDGDGAIGVDEWA  LVQVQGEVDDSVQEARNAEEKAKKA  ALEEAEGTLEHEESKLLR  IEELEEELEAERAAR  LKGTEDELDKYSEALKDAQEKLE  LESDLVQVQGEVDDSVQEARNAEEKAKK  LTENGEFGRQLEEKEA  VMVGMGQKDSYVGDEAQSKRG  LAEKDEEMEQIKRNSQR  LKAGDSDGDGAIGVDEWAVL  MEGDLNEMELQ  VAGDEESYEVFKD  ILEEECMFPK  LTKLEEAEKAADESERGMK  FTPDQMEDYREAFG  ADWKQKYEEGQAELEGSLKEARS  ARIEELEEELEAERAAR  LESDLVQVQGEVDDSVQEARNAEEKAKKA  VQGEVDDSVQEARNAEEKAKKA  IIDQNRDGIISKDDLRD  AAGPAGPSGPRGPAG  IDASERVGL  VASIDDKEELDATDAAIDILG  VAQWRSKYETDAIQRTEELEESKKK  FDMGF  LEEAEGTLEHEESKLLR  VQVQGEVDDSVQEARNAEEKAKKA  VMVGM  ISDLTEQLGETGKS  LTEQLGETGKS  LEDQLSELK  ADSVAELGEQIDNLQR  TEKTEIQSALEEAEGTLEHEESKILRVQ  LEDQLSEIKAKSDENARQ  LEKTIDDLEDELYAQK  LEKSYELPDGQVITIGNERFRCPET  LKGADPEDVIVAA  FSGDEEFPDLS  FTVDDIIQTGVDNPGHPFIMT  IDDLEDELYAQK  LAQESIMDLENDKQQSDEKLKKKDFETSQ  LKKKMEGDLNEMELQ  FVIDQDKSGFIEEDELK  IVLDSGDGVTHNVPVYEGY  LDDAVRAAEDLKEQAAM  VETEKTEIQSALEEAEGTLEHEESK  VIDQDKSGFIEEDELKLF  VAEQELIDASERVGL  VLADWKQKYEEGQAELEGSLKEARS  LQEAEEQIEAVNSK  VALDFENEMAT  LDDAVRAAEDLKEQ  LEKEKSEYKMEIDDLSSNMEAVSKAKGN  VLSGGTTMYPG  IEELEEELEAERAARAK  LKAGDSDGDGKIGVDEFGAM  LEKEKSEYKMEIDDLSSNMEA  SAPKIPDGEKVDFDDIQKKRQNKD  IVLDSGDGVTHNVPVYEGYALPHA  LVDASERVGL  MGQKDSYVGDEAQSKRG  LEEISERLEEAGGATSAQ  VGMGQKDSYVGDEAQSKRG  LKGGDDLDPAYV  MEIDDLSSNMEA  LREQFEEEQEAKAE  ILGNPSDEDMNSKRVDFEG  LMSL  IEELEEELEAERAARAKVEKQRAD  VMDLENDKQQSDEKLKKKDFETSQL  LADWKQKYEEGQAELEGS  LRGDSGPAGPPGEQGM  LDDVIQTGVDNPGHPF  VQHELEEAEERADIAETQ  LQEAEEQIEAVNSKCAS  LGAPGPSGAPGPVGPAGKTGDRGETGPAG  VIDQDKSGFIEEDELK  LKAGDSDGDGAIGVDEWAV | 2.38  2.28  2.23  1.46  1.27  1.00  0.94  0.74  0.72  0.72  0.71  0.71  0.68  0.67  0.66  0.65  0.65  0.63  0.61  0.60  0.58  0.56  0.54  0.53  0.53  0.53  0.53  0.52  0.51  0.51  0.51  0.50  0.49  0.48  0.47  0.46  0.46  0.44  0.43  0.43  0.43  0.41  0.40  0.39  0.38  0.38  0.36  0.35  0.35  0.35  0.35  0.33  0.33  0.33  0.33  0.32  0.32  0.32  0.32  0.31  0.31  0.31  0.31  0.31  0.31  0.31  0.30  0.30  0.29  0.29  0.29  0.28  0.28  0.28  0.28  0.28  0.27  0.27  0.26  0.26  0.26  0.26  0.26  0.25  0.24  0.23  0.23  0.22  0.22  0.22  0.22  0.21  0.21  0.21  0.21  0.21 | 0.38  0.33  0.38  0.33  0.48  0.37  0.34  0.30  0.51  0.42  0.41  0.26  0.36  0.31  0.46  0.36  0.37  0.42  0.35  0.34  0.47  0.37  0.34  0.23  0.40  0.46  0.36  0.39  0.38  0.51  0.45  0.29  0.41  0.32  0.39  0.24  0.39  0.30  0.49  0.31  0.35  0.27  0.43  0.45  0.35  0.40  0.40  0.38  0.34  0.41  0.41  0.23  0.35  0.34  0.38  0.43  0.35  0.40  0.25  0.34  0.38  0.57  0.34  0.23  0.36  0.28  0.37  0.32  0.39  0.33  0.22  0.45  0.41  0.36  0.29  0.32  0.51  0.34  0.44  0.38  0.45  0.38  0.26  0.40  0.31  0.34  0.36  0.27  0.45  0.52  0.39  0.36  0.33  0.49  0.37  0.35 | 0.19  0.17  0.19  0.15  0.23  0.13  0.23  0.15  0.25  0.28  0.21  0.18  0.20  0.21  0.31  0.20  0.25  0.20  0.19  0.11  0.22  0.21  0.16  0.11  0.21  0.13  0.21  0.17  0.27  0.21  0.23  0.17  0.23  0.17  0.19  0.11  0.14  0.22  0.22  0.21  0.21  0.15  0.26  0.21  0.12  0.22  0.22  0.22  0.25  0.23  0.13  0.17  0.22  0.13  0.19  0.23  0.24  0.27  0.16  0.24  0.20  0.23  0.19  0.20  0.19  0.20  0.14  0.19  0.22  0.22  0.12  0.20  0.17  0.18  0.12  0.18  0.17  0.20  0.20  0.19  0.16  0.21  0.25  0.22  0.24  0.27  0.14  0.16  0.18  0.27  0.21  0.18  0.18  0.17  0.22  0.18 | AGDDAPRAVFPS  GPAGPSGPRGPAGIA  AGPAGPSGPRGPAGIA  VAPEEHPTLL  GFAGDDAPRAVFPS  GAAGPAGPSGPRGPAGIA  FLGM  RVAPEEHPTLL  ELPDGQVITIG  KSYELPDGQVITIG  LLPVLYPPVVEE  LGEQIDNL  NWDDME  RGDSGPAGPPGEQGML  FETF  GSAGPRGPSGNIGMPGMTGPQ  TIIDQNRDGIIS  GEKLKGADPEDVIVA  SGSAGKDGMSGLPGPSGPSGPRGR  SGPGGPTGPSGM  GEQIDNL  ELPDGQVI  FLPM  KSYELPDGQVITI  TGPIGPPGSGGAPGDKGE  FAGDDAPRAVFPS  SNKLLPVLYPPVVEE  GSAGPRGPSGNIGMPGMTGPQGE  NPPKYDKIEDM  GFNPPDLDIM  LRVAPEEHPTL  GVDNPGHPF  GWLDKNKDPLNDSVVQ  KDLLDPIISDR  KSYELPDGQVI  GSSGPGGERGPPGPMGPS  ELPDGQVITI  AGFAGDDAPRAVFPS  EWAVL  LDFE  EASGPINF  SAGKDGMSGLPGPSGPSGPRGR  FVLD  LDFENE  AGPRGPSGNIGMPGMTGPQ  SGPSGPAGF  KDDLRDVLA  KDLLDPIISD  LADL  KLLPVLYPPVVEE  LTDRENQSVL  DLAGRDLTDYLM  GKDGMSGLPGPSGPSGPRGR  MGPAGPPGPPGPPGPPGAPGGGFDM  GSLEDQIIA  GETGKPGEQGLSGEAGAPGPAGS  YYPL  EASGPINFT  FQLPDERGYHIF  DLAGRDLTDY  DLTDY  LLPVLYPPVVE  SPNKGTLDDYVE  KKAEPAPEPVAVPAPK  GDRGVPGDQGLAGPA  GERGEQGPGGPGGF  LPVLYPPVVEE  GAPGGGWVFPAQPL  EIVPGDIVE  GADPEDVIVA  TIVNSPNKGTLDDYVE  TIIDQNRDGIISK  FKILDPEAT  GDDAPRAVFPS  KAPEPAPEPEPAPVEPE  VLPL  FDDLPISEQ  FSGDEEFPDL  GVDNPGHPFIM  VALW  DIDYKPLPF  GDQGPAGSAGPAGPRGPSGS  VDTF  VLYPPVVEE  NKLLPVLYPPVVEE  EKTIDDLEDE  GPPGPPGPPGPPGAPGGGFDM  GSAGPRGPSGNIGMPGMTGPQGEAGRE  LEPVLVDIS  LDKVLPVE  DFVEEDELKLF  TGDGVNDAPALK  IIDQNRDGIIS | 3.13  2.64  1.90  1.87  1.75  1.62  1.53  1.42  1.34  1.21  1.09  1.03  0.94  0.90  0.85  0.84  0.76  0.70  0.67  0.67  0.67  0.66  0.63  0.63  0.61  0.61  0.57  0.56  0.56  0.56  0.52  0.49  0.48  0.47  0.46  0.42  0.40  0.38  0.38  0.37  0.36  0.36  0.34  0.34  0.33  0.33  0.32  0.32  0.32  0.32  0.32  0.31  0.31  0.31  0.31  0.30  0.30  0.29  0.28  0.28  0.28  0.28  0.28  0.28  0.28  0.26  0.26  0.26  0.25  0.25  0.25  0.25  0.24  0.23  0.23  0.23  0.23  0.22  0.22  0.22  0.21  0.21  0.21  0.21  0.21  0.20  0.20  0.20  0.20  0.19  0.19  0.19  0.19 | 0.41  0.49  0.51  0.56  0.51  0.49  0.43  0.51  0.31  0.35  0.64  0.35  0.40  0.51  0.40  0.48  0.29  0.38  0.55  0.50  0.34  0.36  0.46  0.44  0.47  0.50  0.53  0.44  0.37  0.39  0.45  0.56  0.28  0.33  0.45  0.61  0.33  0.48  0.42  0.36  0.42  0.56  0.36  0.37  0.47  0.44  0.29  0.33  0.34  0.61  0.32  0.35  0.49  0.68  0.30  0.37  0.59  0.41  0.51  0.35  0.39  0.61  0.34  0.46  0.44  0.52  0.63  0.57  0.39  0.32  0.37  0.29  0.35  0.40  0.52  0.42  0.37  0.43  0.59  0.43  0.50  0.50  0.35  0.52  0.60  0.28  0.56  0.45  0.29  0.35  0.38  0.36  0.29 | 0.24  0.21  0.21  0.26  0.25  0.20  0.28  0.26  0.20  0.21  0.21  0.22  0.21  0.29  0.25  0.23  0.20  0.17  0.25  0.23  0.22  0.25  0.31  0.23  0.22  0.25  0.21  0.23  0.18  0.25  0.25  0.28  0.23  0.25  0.24  0.23  0.20  0.23  0.21  0.27  0.27  0.26  0.25  0.27  0.22  0.28  0.21  0.26  0.26  0.20  0.23  0.21  0.25  0.31  0.22  0.23  0.23  0.26  0.22  0.19  0.24  0.21  0.18  0.21  0.23  0.23  0.21  0.20  0.22  0.20  0.20  0.19  0.23  0.25  0.26  0.29  0.30  0.23  0.28  0.23  0.24  0.24  0.24  0.21  0.19  0.20  0.26  0.20  0.22  0.23  0.24  0.26  0.19 | VAPEEHPTL  VLYPPVVEE  IIDQNRDGIIS  ELPDGQVIT  VIDQDKSGFIE  IIAPPERKYS  FLPM  LEDQLSELK  GVDNPGHPF  GADPEDVIVA  AAGPAGPSGPRGPAG  AGDDAPRAVFPS  LDKNKDPLNDSVVQ  FAGDDAPRA  ELPDGQVITI  KSYELPDGQVITIG  LDFENE  GFNPPDLDIM  ELPDGQVI  LDLL  LMFE  LLVP  MFLE  NPPKYDKIEDM  LLPV  LMF  VLLP  VLF  KSYELPDGQVITI  KSYELPDGQVIT  ELPDGQVITIG  AGPAGPSGPRGPAG  GERGEQGPGGPGGF  SGPGGPTGPSGM  KILDPEAT  VAVL  VLTL  FLGM  GKDGMSGLPGPSGPSGPRGR  FSGDEEFPDLS  GSSGSAGKDGMSGLPGPSGPSGPRGR  EASGPINF  KSYELPDGQVI  KSYELPDGQ  LLLS  FQLPDERGYH  LML  LDFE  LLLT  LVPIVEPE  MFL  SGSAGKDGMSGLPGPSGPSGPRGR  VGPF  RGEQGPGGPGGF  MLL  TGPIGPPGSGGAPGDKGE  LRGDSGPAGPPGEQGM  KAPEPAPEPEPAPVEPE  GPPGPPGPPGPPGAPGGGFDM  LLLP  LLLE  MLF  NWDDME  IVPGDIVE  GSSGPGGERGPPGPMGPSG  FVLD  GLPGPSGPSGPRGR  GEKLKGADPEDVIVA  FETF  EFTF  FLFD  VLYPPVVE  DFPM  GSLEDQIIA  LLTL  VLDL  SAGKDGMSGLPGPSGPSGPRGR  LGVL  IIDQDKSDFVEEDELK  LLPVLYPPVVEE  LGETNPADSKPGSIR  FDDLPISEQ  VLTLG  LTDRENQSVL  VWLG  LLWE  IGPPGSGGAPGDKGE  AINDPFIDL  SAPKIPDGEKV  LNEDKLKDKINE  LGEQIDNL  MGPAGPPGPPGPPGPPGAPGGGFDM  IIDQNRDGIISK  GSSGPGGERGPPGPM  NWDDM  SPNKGTLDDYVE | 1.91  1.75  1.45  1.14  1.13  1.11  1.07  1.06  1.00  0.95  0.75  0.71  0.68  0.67  0.67  0.63  0.60  0.60  0.60  0.58  0.57  0.56  0.55  0.55  0.54  0.54  0.54  0.54  0.53  0.51  0.50  0.50  0.49  0.48  0.46  0.46  0.45  0.44  0.44  0.43  0.43  0.41  0.40  0.39  0.38  0.38  0.38  0.38  0.38  0.37  0.35  0.35  0.34  0.34  0.34  0.33  0.33  0.31  0.31  0.30  0.30  0.29  0.29  0.28  0.28  0.28  0.28  0.28  0.27  0.27  0.27  0.27  0.27  0.26  0.26  0.25  0.25  0.25  0.24  0.24  0.24  0.23  0.23  0.23  0.23  0.23  0.23  0.22  0.22  0.22  0.22  0.22  0.21  0.21  0.20  0.20 | 0.51  0.52  0.29  0.36  0.30  0.40  0.46  0.34  0.56  0.32  0.49  0.41  0.29  0.37  0.33  0.35  0.37  0.39  0.36  0.35  0.39  0.41  0.38  0.37  0.41  0.40  0.42  0.38  0.44  0.43  0.31  0.49  0.52  0.50  0.33  0.33  0.35  0.43  0.49  0.43  0.54  0.42  0.45  0.44  0.34  0.44  0.38  0.36  0.36  0.38  0.39  0.55  0.47  0.53  0.38  0.47  0.52  0.52  0.56  0.43  0.37  0.40  0.40  0.36  0.62  0.36  0.49  0.38  0.40  0.39  0.39  0.52  0.43  0.30  0.36  0.34  0.56  0.38  0.31  0.64  0.34  0.37  0.35  0.32  0.49  0.46  0.51  0.38  0.35  0.25  0.35  0.68  0.30  0.52  0.43  0.34 | 0.25  0.21  0.19  0.23  0.19  0.23  0.31  0.25  0.28  0.20  0.22  0.24  0.27  0.22  0.20  0.21  0.27  0.25  0.25  0.28  0.26  0.28  0.26  0.18  0.29  0.27  0.29  0.25  0.23  0.24  0.20  0.22  0.23  0.23  0.26  0.23  0.26  0.28  0.25  0.23  0.22  0.27  0.24  0.23  0.29  0.21  0.28  0.27  0.29  0.22  0.26  0.25  0.27  0.23  0.26  0.22  0.27  0.26  0.26  0.31  0.29  0.26  0.21  0.21  0.23  0.25  0.22  0.17  0.25  0.26  0.27  0.21  0.30  0.22  0.28  0.26  0.26  0.25  0.21  0.21  0.20  0.30  0.24  0.23  0.24  0.26  0.22  0.24  0.21  0.20  0.22  0.31  0.18  0.22  0.21  0.18 |


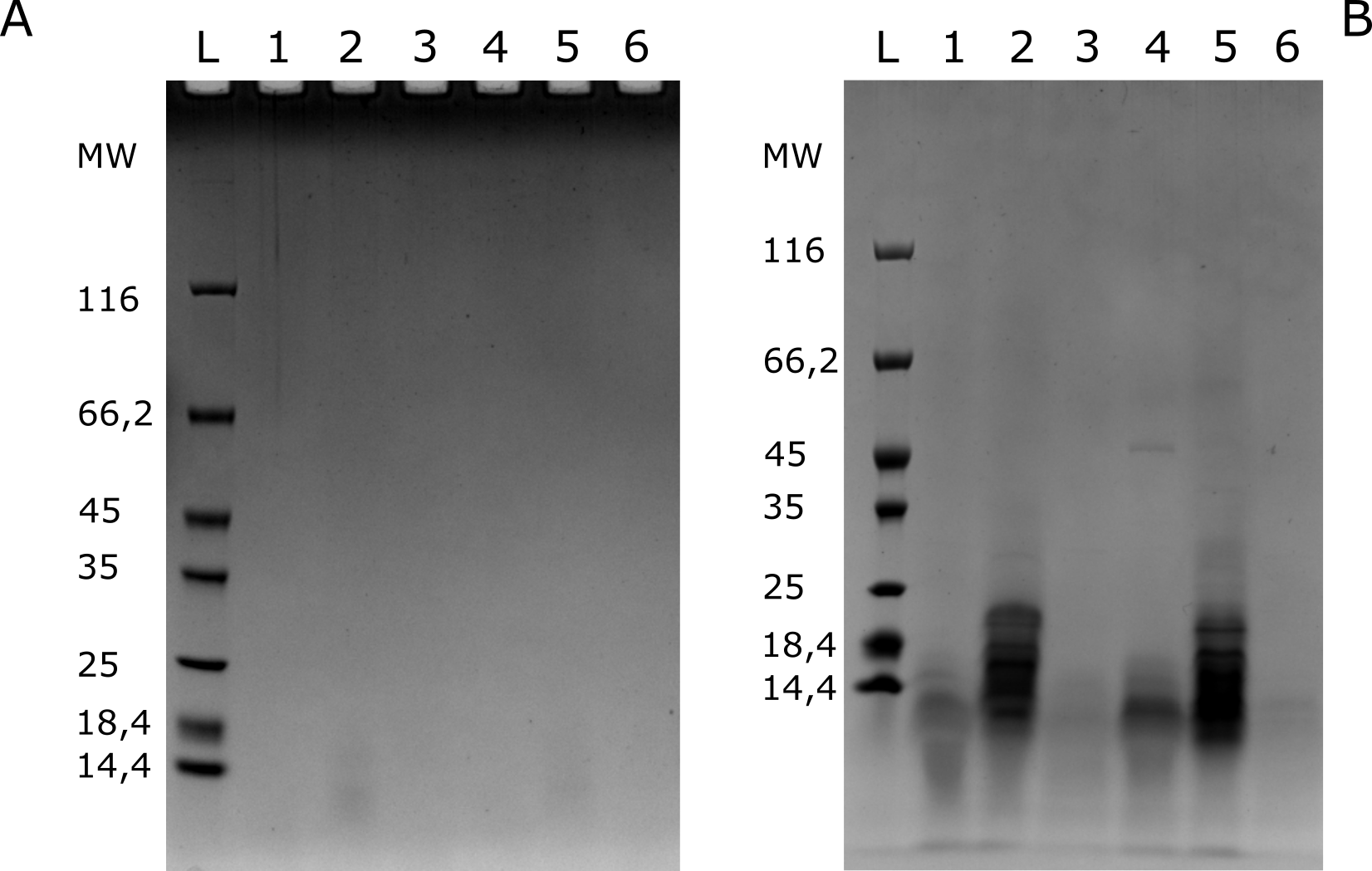


**Figure S1.** SDS-PAGE of FPH samples on 4-20% gradient gels. A: 20 ug protein/peptide (based on Jafarpour et al., 2020). B: 200 ug protein/peptide (based on Jafarpour et al., 2020). L (MW marker), 1 (MCF-Alc), 2 (MCF-Neut), 3 (MCF-Neut&Alc), 4 (HCM-Alc), 5 (HCM-Neut) and 6 (HCM-Neut&Alc).


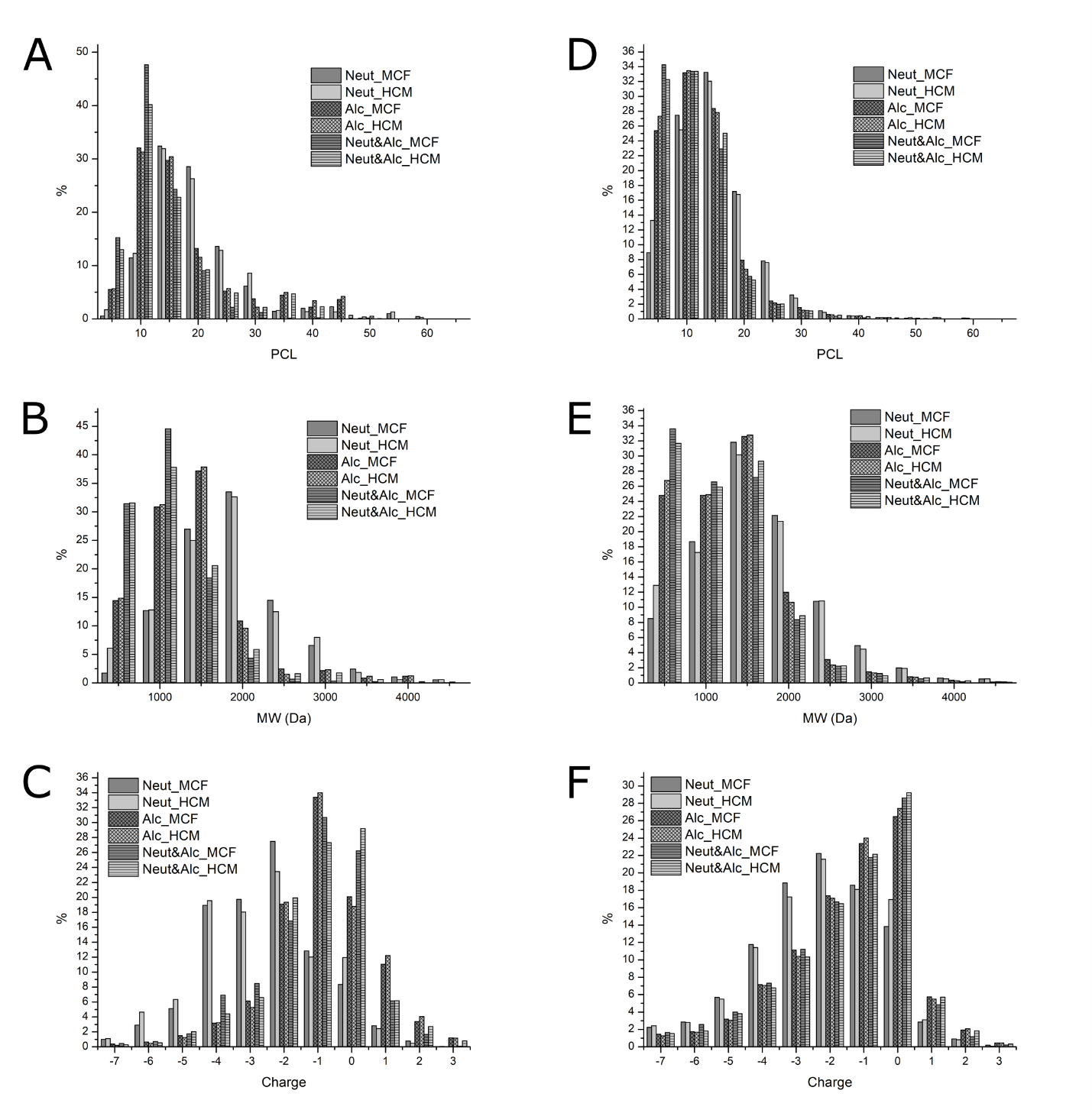


**Figure S2.** Histograms of binned, relative distributions for peptide chain length (A+D), peptide molecular weight (B+E), and peptide charge at pH=7 (C+F) for fish protein hydrolysates obtained from minced cod frame (MCF) and heated cod meat (HCM) by enzymatic hydrolysis with neutrase (Neut), alcalase (Alc) or sequential hydrolysis with neutrase and alcalase (Neut&Alc). Distributions are shown for intensity-weighted data (A-C) and unweighted data (D-F).
